# The complete chloroplast genome sequence of the medicinal plant *Colquhounia coccinea* var. *mollis* (Lamiaceae)

**DOI:** 10.1080/23802359.2019.1666675

**Published:** 2019-09-18

**Authors:** Qi Chen, Dequan Zhang

**Affiliations:** a College of Pharmacy and Chemistry, Dali University, Dali, China;; b Institute of Materia Medica, Dali University, Dali, China

**Keywords:** *Colquhounia coccinea* var. *mollis*, chloroplast, Illumina sequencing, phylogeny

## Abstract

*Colquhounia coccinea* var. *mollis* is a medicinal plant commonly used in Dali area of southwest China. In this study, we sequenced the complete chloroplast (cp) genome sequence of *C. coccinea* var. *mollis* to investigate its phylogenetic relationship in the family Lamiaceae. The total length of the chloroplast genome was 151,965 bp, with 38.4% overall GC content and exhibited typical quadripartite structure, a pair of IRs (inverted repeats) of 25,651 bp was separated by a small single copy (SSC) region of 17,498 bp and a large single copy (LSC) region of 83,165 bp. The cp genome contained 113 genes, including 79 protein-coding genes, 30 tRNA genes, and 4 rRNA genes. The phylogenetic analysis indicated *C. coccinea* var. *mollis* was closely related to *Galeopsis tetrahit* and *Lamium galeobdolon*.

*Colquhounia* Wall. is a small genus in the family Lamiaceae, which only includes six species in the world (Li and Ian 1994). Most of these species are distributed in the Himalayas (Wu 1991). There are five species in China and all of them can be found in Yunnan province (Hu et al. 2012). Among these species, *Colquhounia coccinea* var. *mollis* (Schlechtendal) Prain is widely distributed in Dali area of southwest China which has been used in traditional Chinese medicine for heat-clearing, toning and hemostasis (Editorial Board of National Herbal Compendium 1975). However, for such a medicinal plant, most of the studies focus on describing its chemical compositions and micromorphology (Hu et al. 2012; Li et al. 2013), but nearly no research has been performed for its molecular biology. Here, we reported chloroplast genome sequence of *C. coccinea* var. *mollis* and explored its phylogenetic relationships with related species in the family Lamiaceae.

Fresh and clean leaf materials of *C. coccinea* var. *mollis* were collected from Eryuan county, Yunnan, China (N26.03°, E99.95°); meanwhile, voucher specimen (No. ZDQ126) were collected and deposited at the Herbarium of Medicinal Plants and Crude Drugs of the College of Pharmacy and Chemistry, Dali University. Total genomic DNA was extracted using the improved CTAB method (Doyle 1987; Yang et al. 2014), and sequenced with Illumina Hiseq 2500 (Novogene, Tianjing, China) platform with pair-end (2 × 300 bp) library. About 3.11 Gb of raw reads with 20,623,534 paired-end reads were obtained from high-throughput sequencing. The raw data was filtered using Trimmomatic v.0.32 with default settings (Bolger et al. 2014). Then paired-end reads of clean data were assembled into circular contigs using GetOrganelle.py (Jin et al. 2018). Finally, the cpDNA was annotated by the Dual Organellar Genome Annotator (DOGMA; http://dogma.ccbb.utexas.edu/) (Wyman et al. 2004) and tRNAscan-SE (Lowe & Chan 2016).

The annotated chloroplast genome was submitted to the GenBank with an accession number MN165115. The total length of the chloroplast genome was 151,965 bp, with 38.4% overall GC content. With typical quadripartite structure, a pair of IRs (inverted repeats) of 25,651 bp was separated by a small single copy (SSC) region of 17,498 bp and a large single copy (LSC) region of 83,165 bp. The cp genome contained 113 genes, including 79 protein coding genes, 30 tRNA genes, and 4 rRNA genes. Of these, 17 genes were duplicated in the inverted repeat regions, 16 genes, and 6 tRNA genes contain one intron, while two genes (*ycf3* and *clpP*) have two introns.

To investigate its taxonomic status, a total of 30 cp genome sequences of Lamiaceae species were downloaded from the NCBI database and used for phylogenetic analysis. After using MAFFT V.7.149 for aligning (Katoh and Standley 2013), jModelTest v.2.1.7 (Darriba et al. 2012) was used to determine the best-fitting model for the chloroplast genomes. Then Bayesian inference (BI) was performed by MrBayes v.3.2.6 (Ronquist et al. 2012) with *Tanaecium tetragonolobum* (No. NC_027955) as outgroup. The results showed that *C. coccinea* var. *mollis* was closely related to *Galeopsis tetrahit* and *Lamium galeobdolon* (Figure 1). Meanwhile, the phylogenetic relationship in Lamiaceae was consistent with previous studies and this would be beneficial to developing markers for further studies.

**Figure 1.**
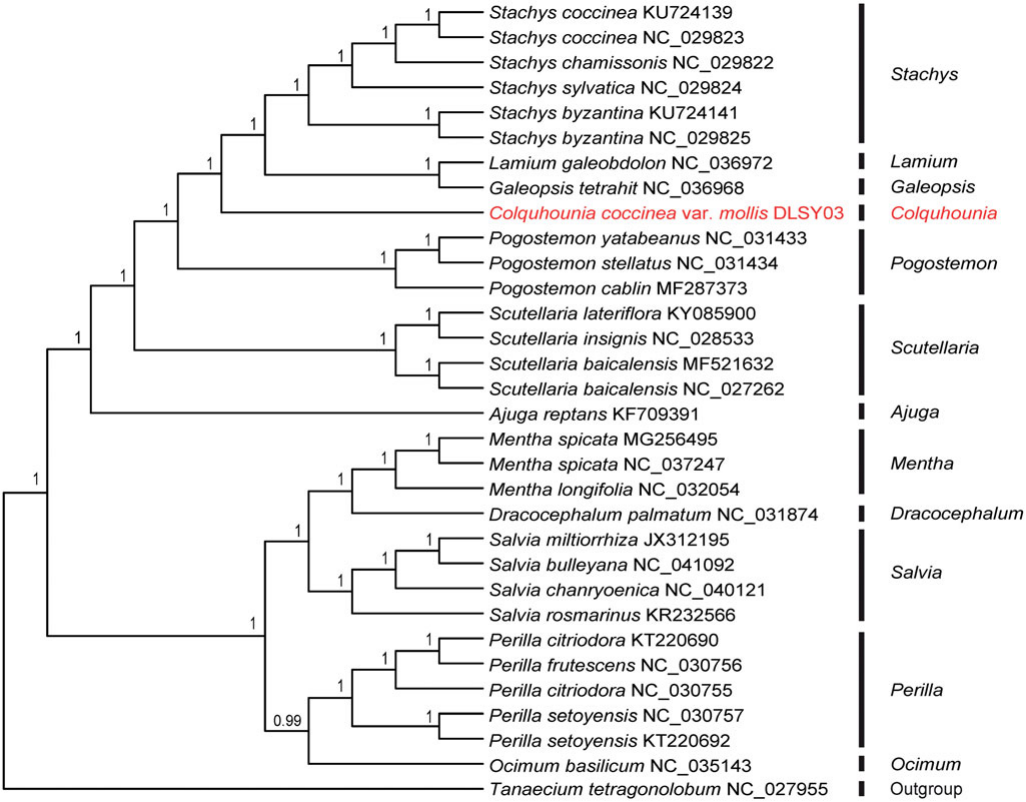
Bayesian inference (BI) tree of 31 species within the family Lamiaceae based on the complete plastome sequences using *Tanaecium tetragonolobum* (No. NC_027955) as outgroup.
